# Connecting MOOSE and NeuroRD through MUSIC: towards a communication framework for multi-scale modeling

**DOI:** 10.1186/1471-2202-12-S1-P77

**Published:** 2011-07-18

**Authors:** Maya Brandi, Ekaterina Brocke, Husain Ahammad Talukdar, Michael Hanke, Upinder S Bhalla, Jeanette Hellgren Kotaleski, Mikael Djurfeldt

**Affiliations:** 1 PDC, Royal Institute of Technology - KTH, Stockholm, S-100 44, Sweden; 2 CSC, Royal Institute of Technology - KTH, Stockholm, S-100 44, Sweden; 3 National Centre for Biological Sciences, Bangalore, India; 4 INCF, Karolinska Institutet - KI , Stockholm, S-171 77, Sweden

## 

The nervous system encompasses structure and phenomena at different spatial and temporal scales from molecule to behavior. In addition, different scales are described by different physical and mathematical formalisms. The dynamics of second messenger pathways can be formulated as stochastic reaction-diffusion systems [1] while the electrical dynamics of the neuronal membrane is often described by compartment models and the Hodgkin-Huxley formalism. In neuroscience, there is an increasing need and interest to study multi-scale phenomena where multiple scales and physical formalisms are covered by a single model. While there exists simulators/frameworks, such as GENESIS and MOOSE [3], which spans such scales (kinetikit/HH-models), most software applications are specialized for a given domain. Here, we report about initial steps towards a framework for multi-scale modeling which builds on the concept of multi-simulations [2]. We aim to provide a standard API and communication framework allowing parallel simulators targeted at different scales and/or different physics to communicate on-line in a cluster environment. Specifically, we show prototype work on simulating electrical activity and Ca^2+^-dynamics in a dendritic spine using MOOSE and NeuroRD [4,8].

Electrical properties of a simple compartment model with soma, dendrite and spine is simulated in MOOSE, while Ca^2+^ dependent reactions and diffusion in the spine is simulated in NeuroRD. In a prototype system, the two simulators are connected using PyMOOSE [5] and JPype [7]. The two models with their different physical properties (membrane currents in MOOSE, molecular biophysics in NeuroRD), are joined into a single model system. Ca^2+^ currents in the electrical model are translated to Ca^2+^ influx rates in NeuroRD, determining the dynamics of the biophysical model. In turn Ca^2+^ dependent events in the spine control properties such as Ca^2+^ dependent ion channels in the electrical model. The joint system, including details of solver methods, is also studied analytically with regard to stability and accuracy and a set of requirements for a generic API allowing parallel solvers to communicate in a multi-simulation is formulated. Experiences from couplers [6] used to couple field models in climate research is taken into consideration. A gap analysis with respect to the existing MUSIC framework [2] is performed.

We demonstrate the interaction of the numerical solvers of two simulators (MOOSE, NeuroRD) targeted at different spatiotemporal scales and different physics while solving a multi-scale problem. We analyze some of the problems that may arise in multi-scale multi-simulations and present requirements for a generic API for parallel solvers. This work represents an initial step towards a flexible modular framework for simulation of large-scale multi-scale multi-physics problems in neuroscience.
